# Resveratrol Ameliorates the Maturation Process of β-Cell-Like Cells Obtained from an Optimized Differentiation Protocol of Human Embryonic Stem Cells

**DOI:** 10.1371/journal.pone.0119904

**Published:** 2015-03-16

**Authors:** Daniela Pezzolla, Javier López-Beas, Christian C. Lachaud, Alejandro Domínguez-Rodríguez, Tarik Smani, Abdelkrim Hmadcha, Bernat Soria

**Affiliations:** 1 Department of Stem Cells, Andalusian Center for Molecular Biology and Regenerative Medicine (CABIMER)—Fundación Progreso y Salud (FPS), Sevilla, Spain; 2 Cardiovascular Pathophysiology, Institute of Biomedicine of Seville (IBIS), Sevilla, Spain; 3 Spanish Biomedical Research Centre in Diabetes and Associated Metabolic Disorders (CIBERDEM), Barcelona, Spain; Universidad Miguel Hernández de Elche, SPAIN

## Abstract

Human embryonic stem cells (hESCs) retain the extraordinary capacity to differentiate into different cell types of an adult organism, including pancreatic β-cells. For this particular lineage, although a lot of effort has been made in the last ten years to achieve an efficient and reproducible differentiation protocol, it was not until recently that this aim was roughly accomplished. Besides, several studies evidenced the impact of resveratrol (RSV) on insulin secretion, even though the mechanism by which this polyphenol potentiates glucose-stimulated insulin secretion (GSIS) is still not clear. The aim of this study was to optimize an efficient differentiation protocol that mimics *in vivo* pancreatic organogenesis and to investigate whether RSV may improve the final maturation step to obtain functional insulin-secreting cells. Our results indicate that treatment of hESCs (HS-181) with activin-A induced definitive endoderm differentiation as detected by the expression of *SOX17* and *FOXA2*. Addition of retinoic acid (RA), Noggin and Cyclopamine promoted pancreatic differentiation as indicated by the expression of the early pancreatic progenitor markers *ISL1*, *NGN3* and *PDX1*. Moreover, during maturation in suspension culture, differentiating cells assembled in islet-like clusters, which expressed specific endocrine markers such as *PDX1*, *SST*, *GCG* and *INS*. Similar results were confirmed with the human induced Pluripotent Stem Cell (hiPSC) line MSUH-001. Finally, differentiation protocols incorporating RSV treatment yielded numerous insulin-positive cells, induced significantly higher PDX1 expression and were able to transiently normalize glycaemia when transplanted in streptozotocin (STZ) induced diabetic mice thus promoting its survival. In conclusion, our strategy allows the efficient differentiation of hESCs into pancreatic endoderm capable of generating β-cell-like cells and demonstrates that RSV improves the maturation process.

## Introduction

Human embryonic stem cells (hESCs) display two important characteristics *self-renewal* and *pluripotency* [1]. Proof-of-concept experiments demonstrate that ESCs have the ability to differentiate into insulin-producing cells, but with a very low efficiency [2–4]. The use of gene selection procedure based on neomycin-resistance transgenes for the insulin and the *Nkx6*.*1* genes allowed the achievement of a purified population that can mature and normalize glycaemia when transplanted in diabetic mice [2,5,6]. Improvement of the *in vitro* differentiation process has benefited from a deeper knowledge of islet development. Sequential expression of the transcription factors [7–9] and signaling pathways [10] involved in human β-cell genesis are instrumental to achieve *in vitro* differentiation processes. Hence, the common approach to differentiate hESCs is based on a multi-stages protocol attempting to reproduce *in vivo* pancreas development aiming to induce hESCs to follow a sequential transition through mesendoderm, definitive endoderm, gut-tube endoderm, pancreatic endoderm and endocrine precursor stages, finally obtaining functional insulin-expressing cells [11–13].

The major problems in directing hESCs differentiation to β-cell-like cells are the low reproducibility of the current differentiation protocols and the low amount of insulin-secreting cells obtained at the end of the differentiation processes. Protocols described so far generate *PDX1* and/or insulin positive cells, which need further maturation when transplanted into immunocompromised mice [14–16]. Maturating endocrine precursors toward specialized and functional hormone-secreting cells, still the most problematic step for hESCs differentiation to insulin-producing cells [17,18]. Despite the great number of biologically active compounds that have been already tested for this purpose, none of them has successfully worked [19,20]. Cells obtained from *in vitro* differentiation strategies are not mature enough to be completely functional; although they express different markers of β-cells, such as insulin, GLUT2 or GK, they could show functional problems due to impairment of the glucose sensing pathway or the exocytotic machinery [21–24]. Hence, strategies to ameliorate the *in vitro* maturation process of endocrine precursors are needed and up quite recently were achieved [12,13].

On the other hand, several studies reported the beneficial impact of resveratrol (RSV) on insulin secretion and how this compound potentiates glucose-stimulated insulin secretion (GSIS), not only in rat insulinoma cell lines (INS-1E), but also in isolated human islets [25]. On this basis, we investigated whether RSV could improve the final maturation step of hESCs differentiation towards β-cells. RSV (3,5,4′-trihydroxy-trans-stilbene) is a polyphenol that has been shown to activate SIRT1, a NAD^+^-dependent deacetylase [26,27]. We have recently shown that SIRT1 contributes to the establishment of specific developmental/differentiation programs of hESCs [28]. Other studies demonstrated the effect of RSV on insulin secretion using INS-1E and human islet [25,29]. SIRT1 represses mitochondrial uncoupling protein 2 (*Ucp2*) transcription by binding directly to its promoter [30], resulting in increased ATP production and insulin secretion in INS-1E and in BESTO mice islets [31,32]. Additionally, RSV induced an up-regulation of key genes for β-cell function such as *Pdx1*, *Glut2*, *Gk*, *Hnf1α* and *Tfam* in both INS-1E cells and human islets [25], this up-regulation has been described as a possible mechanism by which RSV potentiates metabolism-secretion coupling in β-cells and interestingly for the maintenance of the β-cell identity [33,34]. In the present study, we showed for the first time that RSV is a critical compound improving the maturation of hESCs-derived endocrine precursors towards insulin-secreting cells, thus proposing its use for a more efficient insulin-secreting cells differentiation strategy.

## Results

### Effects of resveratrol on insulin content and secretion in INS-1E cells

**Fig 1:**
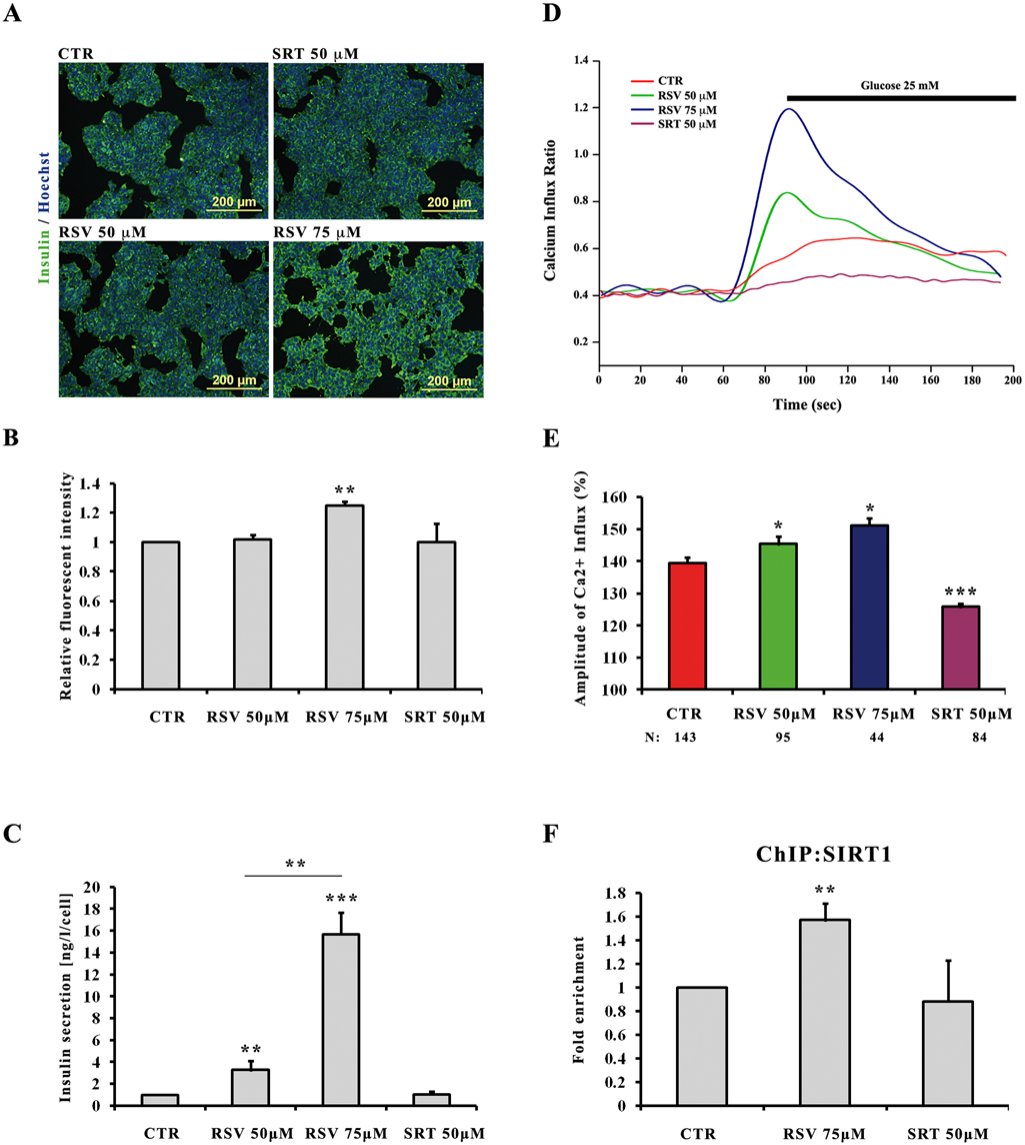
Effects of resveratrol and sirtinol on insulin content and secretion in INS-1E cells. INS-1E cells were cultured under control conditions (CTR) or with the indicated concentrations of RSV and SRT in standard medium during 48 h. **A**: Immunofluorescence images showing representative insulin detection (*green*) after treatments with RSV and SRT. Nuclei are stained with Hoechst (*blue*). **B**: Quantification of insulin staining by MetaMorph analysis. Values are mean ±SE of 2 independent experiments. (**) *p<0*.*01*. **C**: Effects of RSV and SRT on insulin secretion. Insulin release was measured over a 30 min incubation period at a stimulatory glucose concentration of 20 mM. Values are mean ±SE of 4 independent experiments. (**) *p<0*.*01*, (***) *p<0*.*001*. **D**: Representative traces showing the changes in intracellular Ca^2+^ concentration (presented as the ratio of fluorescence at 340 to 380 nm (F340/F380) in Fura-2-loaded cells. **E**: Average of amplitude of RSV and SRT induced Ca^2+^ influx (ratio ±SE), (*) *p<0*.*05*, (***) *p<0*.*001*. Then number of single cells analyzed for each condition is shown below each bar (N). **F**: ChiP assay of SIRT1 binding to *Ucp2* promoter. Chromatin immunoprecipitation was carried out using SIRT1 antibody and it was analyzed by q-PCR of a regulatory region (*Intron2*) in the *Ucp2* promoter. Values are mean ±SE of 3 independent experiments. (**) *p<0*.*01*.

INS-1E cells were treated with different concentrations of RSV (50–75 μM) or with sirtinol (SRT)-SIRT1 inhibitor- 50 μM during 48 hours, and their insulin content and secretion was then analyzed. Comparative immunofluorescence analysis indicated increased insulin content in INS-1E cells treated with 75 μM RSV compared to all other conditions (Fig. 1A). MetaMorph-based fluorescence signals quantification confirmed a 25% increase in insulin expression level in cells treated with 75 μM of RSV compared to control cells; however cells treated with 50 μM RSV or SRT showed no significant changes in insulin content (Fig. 1B). INS-1E cells pre-treated or not with RSV were challenged with 20 mM glucose and then insulin secretion was quantified using ELISA assay. INS-1E treated with 50 and 75 μM of RSV increased their insulin secretion by 3,2 and 15,6 fold respectively, compared to values found in glucose stimulated control cells (Fig. 1C). To test whether RSV treatment of the INS-1E cells could increase intracellular Ca^2+^ concentration upon glucose stimulation, we monitored the dynamic changes in cytosolic free Ca^2+^ using Fura-2AM loaded cells. Measurements of Ca^2+^ influx indicated that intracellular Ca^2+^ concentration was increased in RSV-treated cells in a dose-dependent manner (Figs. 1D and 1E). Quantification of the average maximal amplitude of Ca^2+^ influx (peak Δ ratio) indicated how RSV pre-treated cells displayed significantly higher intracellular Ca^2+^ mobilization than untreated cells. By contrast, and as expected, SRT pre-treatment reduced Ca^2+^ influx levels, which were much lower than those recorded in untreated cells (Fig. 1E). Taking into account these results, the rest of the study was carried out using the most effective RSV concentration of 75 μM.

To shed light on a possible mechanism of action, we checked if the effect of RSV on insulin secretion was related to SIRT1 binding to *Ucp2* promoter and inhibiting its expression, thus causing a better sensing of glucose-stimulated insulin secretion. ChIP experiments confirmed the binding of SIRT1 to the *Ucp2* promoter in INS-1E cells and showed that this binding was significantly increased in cells pre-treated for 48 hours with RSV (Fig. 1F). Based on these results, we decided to investigate the effect of RSV on hESCs and hiPSCs differentiation towards insulin-producing cells.

### Recapitulation of pancreas organogenesis for efficient hESCs differentiation

**Fig 2:**
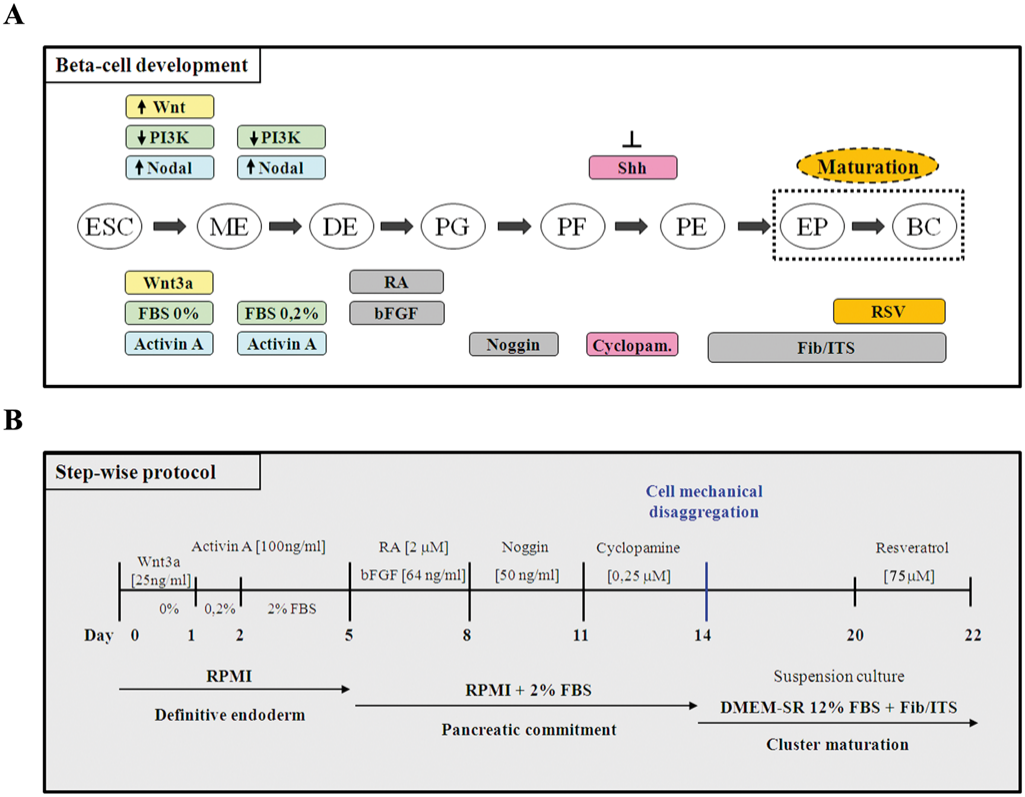
Recapitulation of pancreas organogenesis for efficient hESCs differentiation. **A**: Schematic representation of the steps involved in ESCs differentiation toward a β-cell fate and the factors and signaling pathways involved in this process. ESC, embryonic stem cells; ME, mesendoderm; DE, definitive endoderm; PG, primitive gut; PF, posterior foregut; PE, pancreatic endoderm; EP, endocrine precursors; BC, β-cells. **B**: Schematic representation summary of the step-wise differentiation protocol used to obtain hESC-derived insulin-producing cells. RA, retinoic acid; Fib, fibronectin; ITS, insulin-transferrin-selenium.

**Fig 3:**
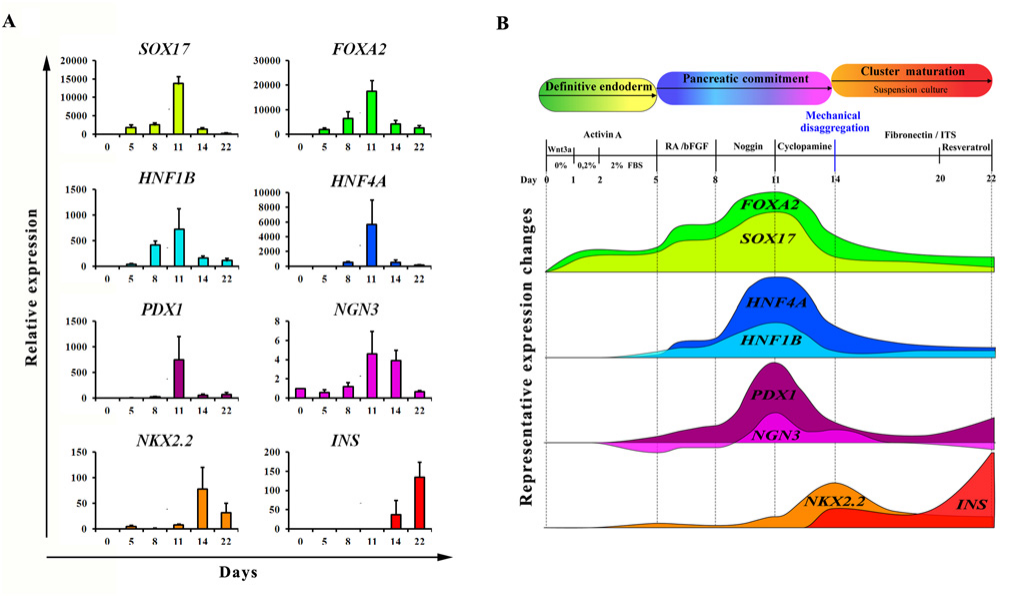
Temporal dynamics of gene expression during hESCs differentiation. **A**: HS181 cells were differentiated to β-cell-like cells as described above. Cell samples were collected at days 0, 5, 8, 11, 14 and 22 and were analyzed by q-PCR for *SOX17*, *FOXA2*, *HNF1B*, *HNF4A*, *PDX1*, *NGN3*, *NKX2*.*2* and *INS* gene expression. For each sample, relative expression was normalized to day 0. **B**: Overview of the results obtained from q-PCR analysis of the genes described above. The schematic representation shows the sequential expression and temporal variation of these genes during our differentiation protocol.

We sought to recapitulate all the factors and signaling pathways involved in β-cell formation during organogenesis (Fig. 2A) in order to develop a highly efficient step-wise protocol, as schematically described in Fig. 2B. To corroborate the efficiency of our differentiation protocol, we analyzed the temporal expression of some key transcription factors involved in pancreas organogenesis at different time-points during differentiation protocol. As shown in Fig. 3A, *SOX17* and *FOXA2* were detected after five days of culture, indicating the differentiation of hESCs to a definitive endoderm population. At the same time the expression of *HNF1B* and *HNF4A*, two factors respectively essential for *NGN3* induction and hepatic formation, was increased. Their up-regulation decreased later in the differentiation protocol at day 14, indicating a pancreatic endoderm specification at the expense of other foregut endoderm lineages. The expression of *PDX1* turned on showing a peak at day 11, then decreased during endocrine proliferation at day 14 and finally was re-expressed on mature β-cell-like cells. The step of endocrine induction occurred between days 11–14 as evidenced by the peak of expression of the endocrine progenitor marker *NGN3* that started to decrease at day 14 in conjunction with the high expression of the β-cell precursor marker *NKX2*.*2*. Insulin, a late marker of pancreatic endocrine differentiation was first detected at day 14 and drastically increased at the end of the differentiation protocol (day 22). The graphical overview of the dynamic expression of the studied genes (Fig. 3B) allows a better appreciation of the different and sequential pancreatic developmental stages that could be successfully recapitulated by our multi-steps differentiation protocol.

### Characterization of β-cell-like cells derived from hESCs differentiation

**Fig 4:**
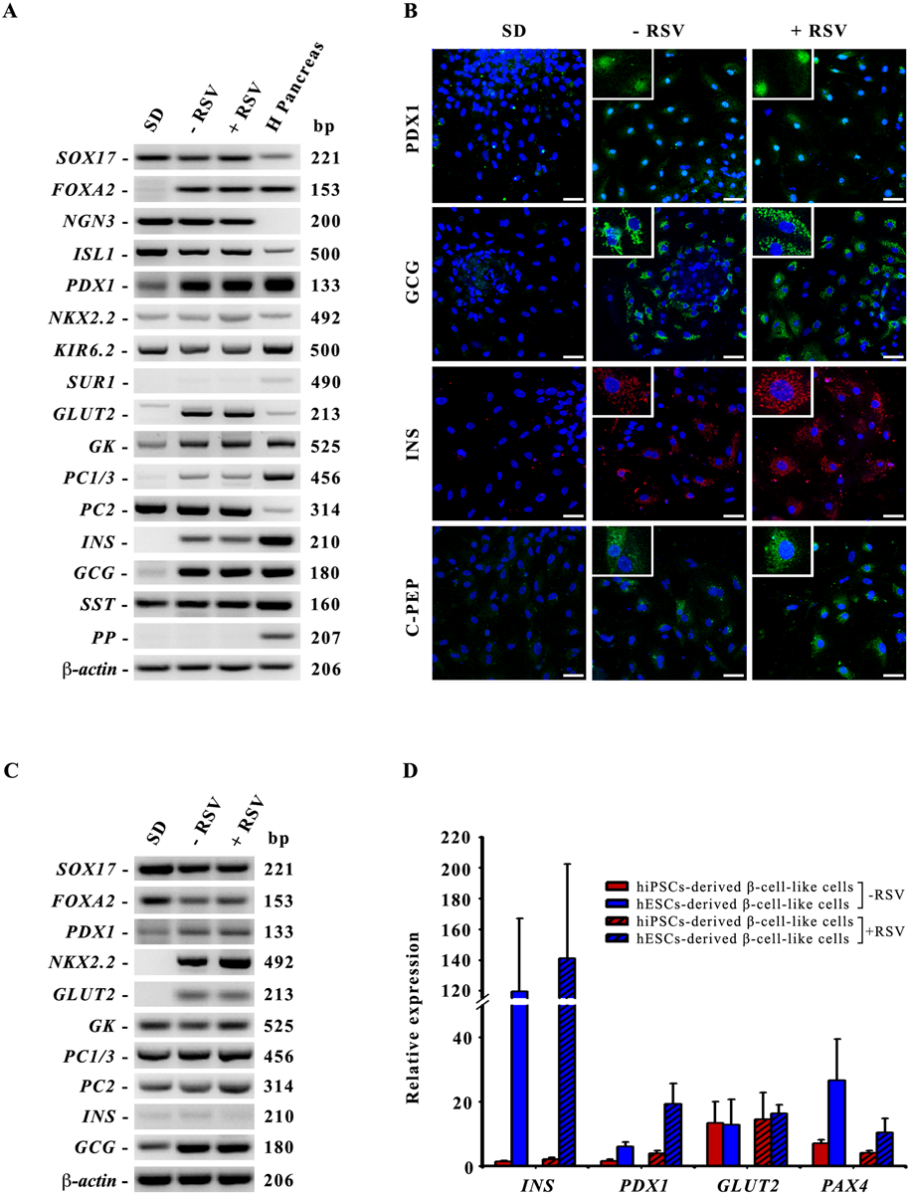
Characterization of the differentiated islet-like clusters obtained at the end of the differentiation protocol. **A**: RT-PCR detection of definitive endoderm, endocrine precursors and islet cells specific markers. β-actin was used as the input control. SD: hESCs spontaneously differentiated; -RSV: hESCs subjected to our differentiation protocol without RSV addition; +RSV: hESCs subjected to our differentiation protocol with RSV addition. H Pancreas: human pancreas as positive control. **B**: Immunofluorescence analysis of PDX1, GCG, INS and C-PEP expression in spontaneous differentiated cells (SD) and in differentiated β-cell-like cells without (-RSV) or with RSV addition (+RSV). Nuclei are stained with Hoechst (*blue*). Scale bar 50 μm. **C**: RT-PCR detection of definitive endoderm, endocrine precursors and islet cells specific markers of hiPSCs-derived β-cell-like cells. β-actin was used as the input control. **D**: q-PCR comparison of the levels of expression of some β-markers in β-cell-like cells derived from hiPSCs (*red bars*) or hESCs (*blue bars*) without or with RSV treatment (-RSV, +RSV).

Given the effects of RSV observed in INS-1E cells we investigated if it could similarly improve the insulin secretion of hESCs-derived β-cell-like cells. hESCs were subjected to the differentiation with or without RSV (75 μM) addition during the last two days of differentiation and they were finally characterized for the expression of β-cell markers. We examined a panel of islet cell specific marker genes, such as *PDX1*, *GLUT2*, *ISL1*, *NGN3*, *NKX2*.*2*, *GK*, *PC1/3*, *GCG*, *SST* and *INS* which were all clearly detected by RT-PCR in both conditions (-RSV/+RSV) (Fig. 4A). The mature endocrine cells markers PDX1, GCG, INS and C-PEP were also analyzed by immunofluorescence (Fig. 4B). These markers could be detected in many of both RSV-treated and untreated cells, being insulin immunostaining levels higher in the RSV-treated condition. Similar results were obtained by applying this differentiation protocol to the hiPSC line MSUH-001. As shown in Fig. 4C, RT-PCR analysis of hiPSCs at the end of the differentiation protocol could confirm the expression of definitive endoderm markers (*SOX17*, *FOXA2*), endocrine precursor markers (*PDX1*, *NKX2*.*2*) and islet-specific markers (*GLUT2*, *GK*, *PC1/3*, *PC2*, *INS* and *GCG*). These results confirmed the efficacy and reproducibility of our optimized differentiation protocol for the achievement of β-cell-like cells both from hESCs and hiPSCs. Nevertheless, the efficiency of the differentiation protocol is not the same for hiPSCs, comparing by q-PCR analysis the expression levels of some β-markers we observed that insulin expression is extremely lower in hiPSCs-derived β-cell-like cells compared to hESCs-derived cells, as well as *PDX1* and *PAX4* expression (Fig. 4D).

### Effects of resveratrol on maturation of hESCs derived β-cell-like cells

**Fig 5:**
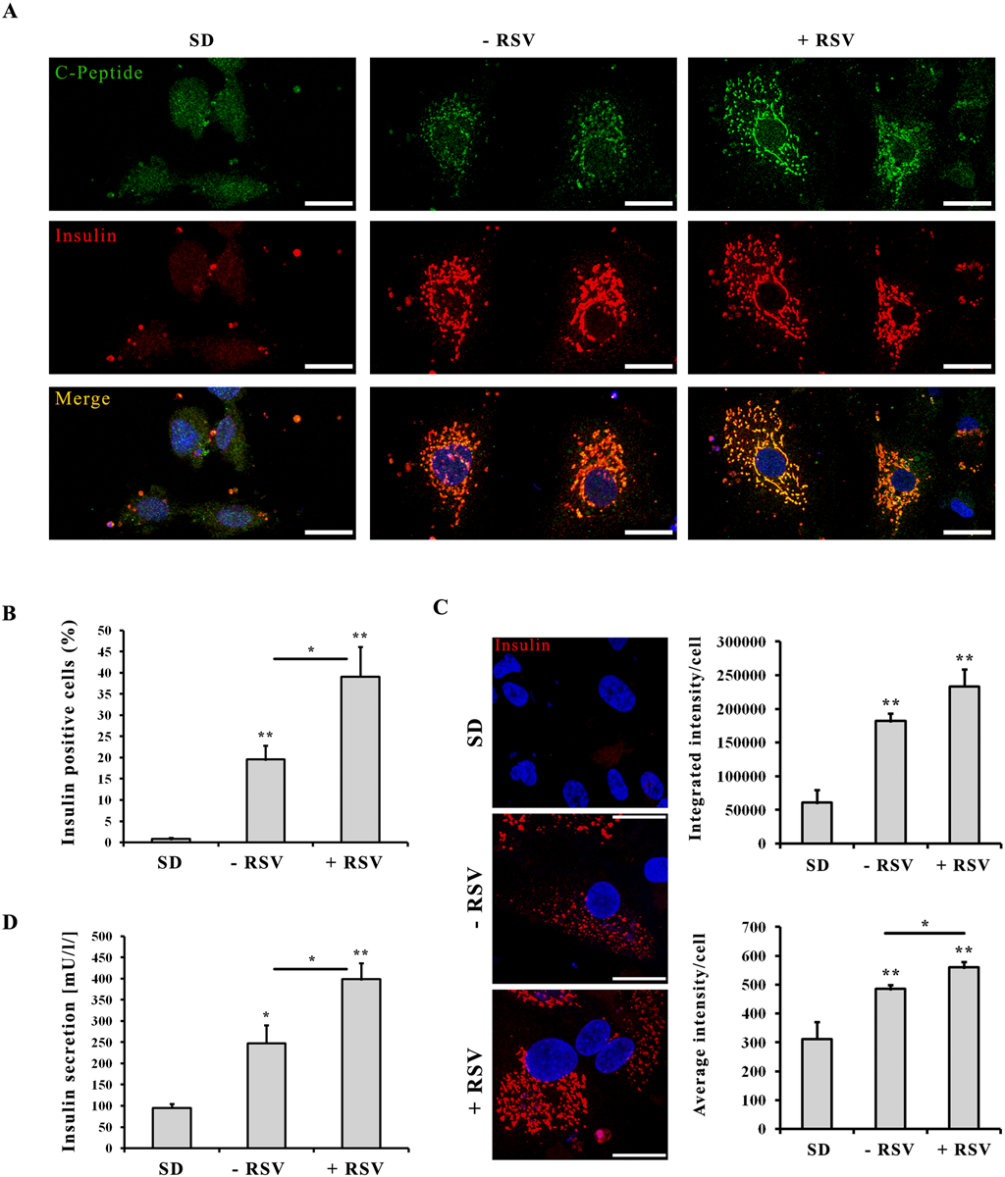
Effects of resveratrol on maturation of hESCs-derived insulin-secreting cells. **A**: Comparative confocal immunofluorescence expression of insulin (*red*) and C-peptide (*green*) expression in hESCs spontaneously differentiated cells (SD) and in differentiated β-cell-like cells without (-RSV) or with (+RSV) RSV addition. Nuclei are stained with Hoechst (*blue*). Scale bar 25 μm. **B**: Quantification by MetaMorph analysis of insulin positive cells (%). **C**: Immunofluorescence analysis of insulin content. Nuclei are stained with Hoechst (*blue*). Scale bar 25 μm. Graphs show the quantification by MetaMorph analysis of Integrated Intensity of insulin-positive cells (upper graph) and Average Intensity of insulin-positive cells (lower graph). Values are mean ±SE of 3 to 4 independent experiments. (*) *p<0*.*05*, (**) *p<0*.*01*. **D**: ELISA quantification of insulin secretion. Insulin release was measured using a Mercodia ELISA kit after 1 hour incubation period with glucose 7 mM. Values are mean ±SE of 3 to 4 independent experiments. (*) *p<0*.*05*, (**) *p<0*.*01*.

To better understand the effects of RSV on cell maturation we further characterized hESCs-derived insulin-producing cells at the final stage of differentiation protocol (Fig. 5). We performed confocal double immunofluorescence analysis of spontaneously differentiated cells (SD) and hESCs derived β-cell-like cells without or with RSV addition (-RSV/+RSV) and we evidenced a perfect colocalization of insulin and C-peptide in cytoplasmic granules of RSV-treated cells (Fig. 5A). MetaMorph-based quantitative analysis of insulin immunofluorescence levels confirmed that RSV-treated cultures displayed a significantly higher number of insulin positive cells compared to RSV-untreated cultures (39,1% vs. 19,6% positive cells, respectively, *p<0*.*05*) (Fig. 5B). In addition, at the single cell level, RSV-treated cells displayed significantly higher insulin levels than RSV-untreated cells (*p<0*.*05*) (Fig. 5C). The results obtained with INS-1E cells indicate that the effect of RSV is principally associated with a more efficient glucose-stimulated insulin secretion. Hence, as expected, differentiated cells treated with RSV (+RSV) showed an increase of 1,6 and 4,2 fold in the secretion of insulin compared with non-treated cells (-RSV) and spontaneous differentiated cells (SD) respectively, as shown in Fig. 5D.

**Fig 6:**
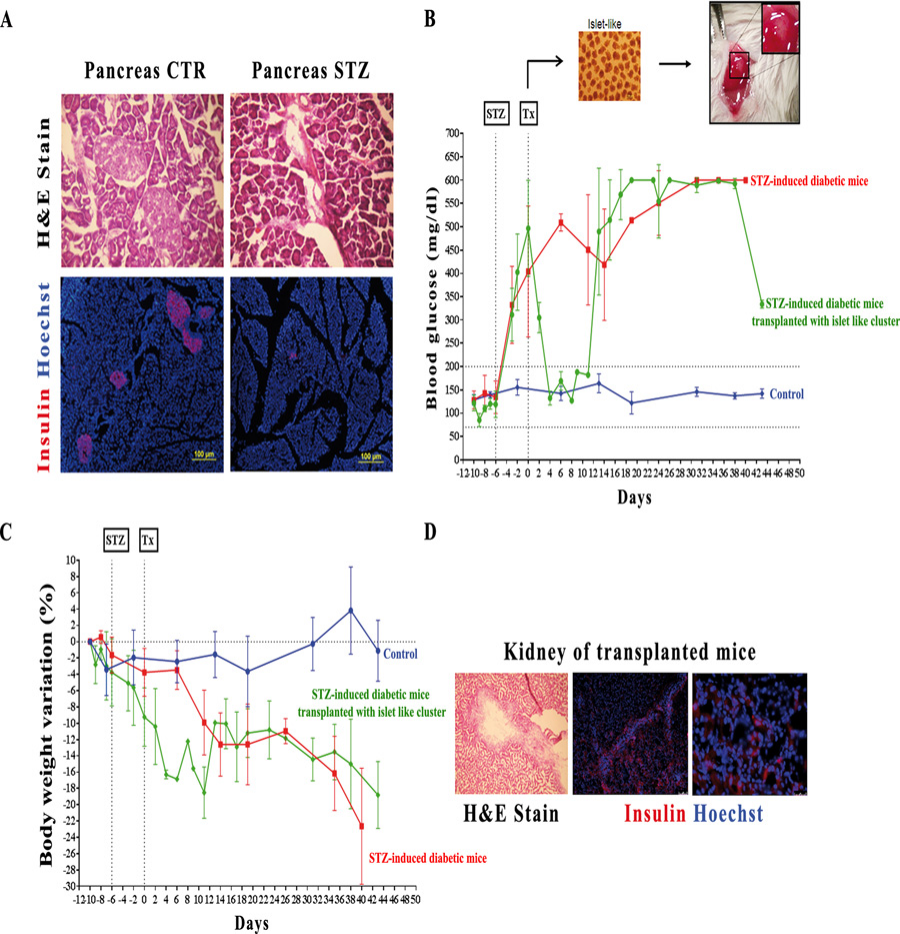
In vitro and in vivo studies of hESCs-derived insulin producing cells functionality. **A**: Immunohistochemical analyses of pancreas sections from control mice (Pancreas CTR) and STZ-induced diabetic mice (Pancreas STZ). Upper panel shows hematoxylin and eosin stain (H&E stain) of Langerhans Islets, which are completely compromised in diabetic mice. Lower panel shows the complete loss of insulin stain (*red*) in diabetic pancreas. **B**: Nonfasting blood glucose measurements of control mice (*blue line*, n = 4), STZ-induced diabetic mice (*red line*, n = 3) and STZ-induced diabetic mice transplanted (Tx) with 2000 islet-like clusters derived from hESCs differentiation (*green line*, n = 3). **C**: Percentage of body weight variations in control mice (*blue line*, n = 4), STZ-induced diabetic mice (*red line*, n = 3) and STZ-induced diabetic mice transplanted with 2000 islet-like clusters derived from hESCs differentiation (*green line*, n = 3). **D**: Immunohistochemical analyses of a transplanted kidney showing H&E stain of the engraftment and a dispersed insulin staining (*red*) throughout the renal parenchyma. Scale bar 75 μm and 25 μm respectively.

To study *in vivo* functionality, differentiated cells treated with RSV were transplanted under the kidney capsule of STZ-induced diabetic NOD/SCID mice with damaged islets of Langerhans (Fig. 6A). Soon after transplantation, we observed a period of normoglycaemia within 8 days, which correlates with a subsequent period of maintenance of the body weight, in contrast with the constant hyperglycaemia and body weight loss observed in non-transplanted diabetic mice (Figs. 6B and 6C). The subsequent regression to a hyperglycaemic state could be ascribed to the inability of maintaining the transplanted cells in the site of engraftment, as shown by the immunohistological stain of the transplanted mice where, even if it is still possible to detect some insulin positive cells throughout the kidney section, they were in a very limited and scattered way (Fig. 6D).

### Mechanism of action of resveratrol

**Fig 7:**
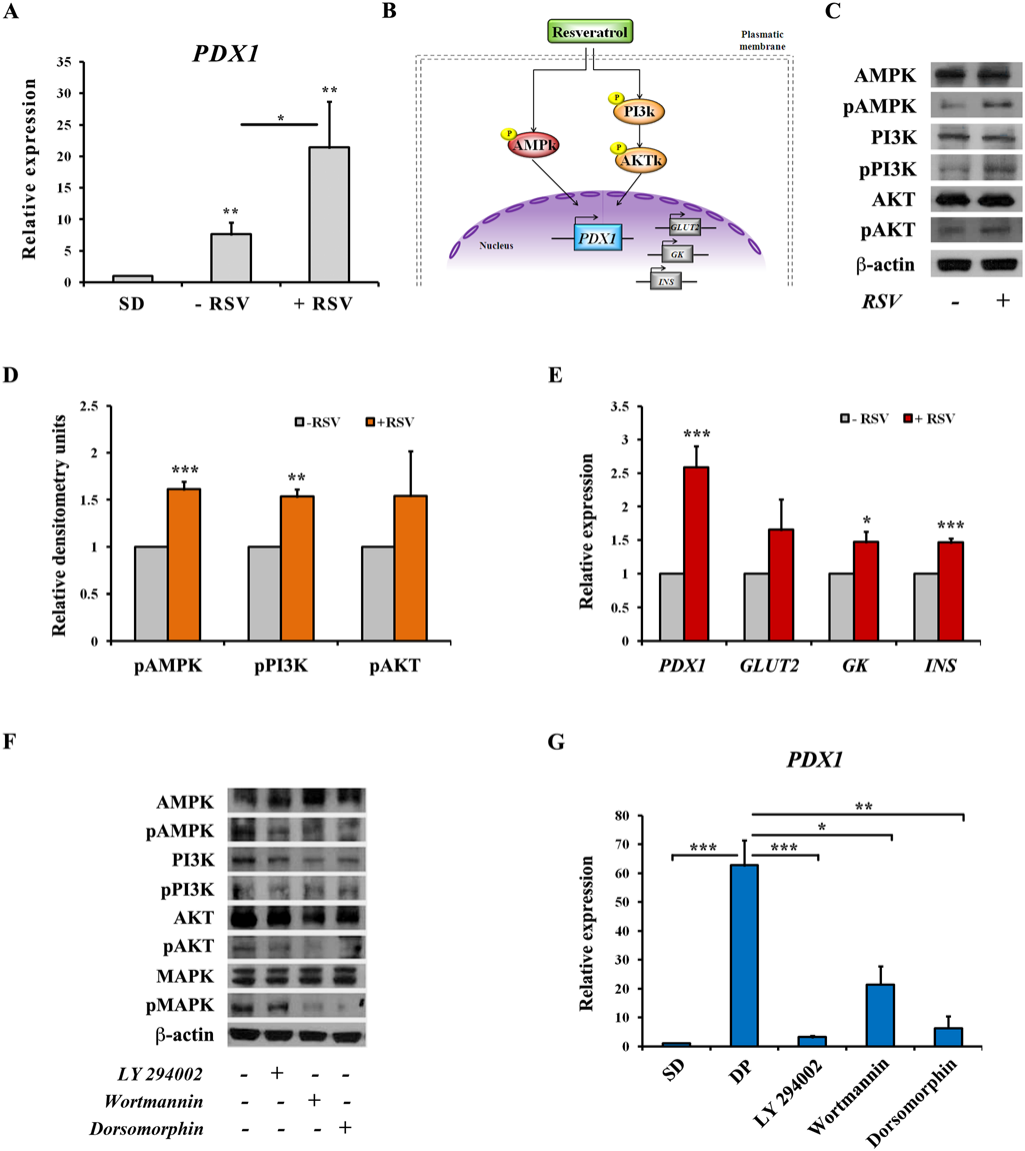
Mechanism of action of resveratrol. **A**: Analysis of *PDX1* expression by q-PCR in hESCs derived insulin-producing cells after RSV treatment. Values are mean ±SE of 5 independent experiments. (*) *p<0*.*05*, (**) *p<0*.*01*. **B**: Schematic representation of the signaling pathways proposed to be the targets of RSV action for the induction of *PDX1* and other specific β-cell genes transcription. **C**: Immunoblotting of hESCs-derived β-cell-like cells extracts treated with (+RSV) or without (-RSV) RSV. Levels of phosphorylated AMPK, PI3K and AKT proteins (pAMPK, pPI3K, pAKT) and total kinases were assessed. β-actin was used as loading control. **D**: Densitometry quantification of phosphorylated proteins normalized to total proteins. Values are mean ±SE of 3–4 independent experiments. (**) *p<0*.*01*, (***) *p<0*.*001*. **E**: Analysis of *PDX1*, *GLUT2*, *GK* and *INS* expression by q-PCR in hESCs derived insulin-producing cells after RSV treatment. Values are mean ±SE of 3–5 independent experiments. (*) *p<0*.*05*, (***) *p<0*.*001*. Results of experiments shown in the panels **F** and **G** are carried out on cells harvested at day 11 of the differentiation protocol; which corresponds with the first peak of *PDX1* expression. **F**: Western blot representing the phosphorylated state of AMPK, PI3K, MAPK and AKT kinases with or without the addition of specific inhibitors. β-actin was used as loading control. **G**: q-PCR analysis of *PDX1* expression after treatment with specific inhibitors of upstream signaling pathways. SD: spontaneous differentiation; DP: cells subjected to the differentiation protocol (until day 11); cells subjected to the differentiation protocol and treated during last 48 hours with specific inhibitors: LY 294002 50 μM, Wortmannin 2 μM and Dorsomorphin 10 μM. Values are mean ±SE of 2 independent experiments. (*) *p<0*.*05*, (**) *p<0*.*01*, (***) *p<0*.*001*.

*PDX1* expression was modulated upon treatment with RSV, which may explain the increase of insulin-positive cells and theire functionality, we performed a q-PCR analysis and we found that it was increased by 3 fold in cells treated with RSV compared to RSV-untreated cells (Fig. 7A). *PDX1* is indeed important to induce the expression of different β-cell genes such as *GLUT2*, *GK* and insulin that characterize a mature and functional cell. Hence, with the aim to understand the mechanism of action of RSV, we studied the phosphorylation status of several kinases involved in the signaling pathways that lead to *PDX1* activation (Fig. 7B). We observed an increase of AMPK phosphorylation in cells treated with RSV compared with non-treated cells and, in the same way, the PI3K/AKT pathway was also found to be stimulated at higher extent in response to RSV treatment, as detected by western blot of the phosphorylated proteins (Figs. 7C and 7D). Thus, indicating that RSV induced activation of the two main signaling pathways involved in *PDX1* transcription by increasing the phosphorylated status of its upstream kinases. As a consequence of this activation, we observed an increased expression of both *PDX1* and its downstream target genes *GLUT2*, *GK* and *INS* (Fig. 7E). We postulated that these two kinase cascades are also responsible for the first *PDX1* induction at day 11 of our differentiation protocol (see Fig. 3) and as expected, inhibition of AMPK or PI3K by specific inhibitors (Fig. 7F) caused a drastic decrease of *PDX1* expression (Fig. 7G).

## Discussion

Differentiation of hESCs into insulin-producing cells is possible as reported by different studies [3,35–39]. Nevertheless, the differentiation protocols published so far only reported the achievement of a low percentage of insulin-secreting cells (from 6% to 25%) [15,24,35,39] being their amount of insulin secretion lower than that displayed by β-cells in healthy Langerhans islets. We established an efficient strategy to differentiate hESCs toward insulin-producing cells and we demonstrated that RSV is a key factor for the induction of a β-like phenotype. Furthermore, the quite recent success in generating mature β-cells from hESC is very encouraging [12,13] and clearly indicates, not only the consistency of the differentiation processes, but also its achievement throughout different groups.

Previous works using different animal models of insulin resistance showed that RSV was able to improve insulin secretion, although the exact mechanism of its action is still poorly elucidated [40–42]. As shown in this work, the increase of insulin secretion caused by RSV treatment, correlated with an increase of Ca^2+^ entrance in INS-1E β-cells, thus demonstrating that RSV was somehow regulating the conventional insulin secretion pathway. Bordone and colleagues [30] showed that SIRT1 overexpression induced *Ucp2* inhibition, thus causing a better coupling of glucose metabolism with insulin secretion. UCP2 is a mitochondrial uncoupling protein that physiologically attenuates glucose-stimulated insulin secretion in β-cells [43]. Using a similar experimental approach we performed ChIP experiments confirming that SIRT1 binds to the *Ucp2* promoter and that RSV, as a SIRT1 activator, could improve this binding. Remarkably, and in agreement with Vetterli et al [25] we could not detect a decrease in *Ucp2* transcription (data not shown) upon SIRT1 activation. This could be due to the rapid and dynamic regulation of *Ucp2* content at both transcriptional and translational levels [44,45], a mechanism, which is necessary for β-cells to respond to fluctuating nutrient supply. The results obtained with INS-1E cells led us to wonder whether RSV would exhibit similar effects on hESCs pancreatic differentiation, with the aim of using the effect of this polyphenol as a new strategy to increase the amount of insulin obtained from hESCs-derived β-cell-like cells. For this purpose, our first goal was to define a reproducible and efficient step-wise pancreatic differentiation protocol based on the developmental phases of pancreas organogenesis. The first step consisted in obtaining an efficient generation of definitive endoderm (DE) from hESCs. This goal has been readily achieved using a TGFβ family member, Activin A, to activate Nodal signaling, and low serum concentration to avoid the activation of PI3K. Wnt3a-mediated Brachyury expression is also important for migration of precursors cells through the anterior region of the primitive streak (PS) and the formation of a mesendoderm population from which both endoderm and mesoderm will emerge depending on the magnitude and duration of Nodal signaling [46–49]. Hence, exposing hESCs to a combination of Activin A and Wnt3a in a media with low serum concentration generated definitive endoderm as reported by D’Amour [50]. The following step consisted on triggering DE to foregut patterning, which results from the complex cross talk between mesoderm and endoderm, involving gradients of fibroblast growth factors (FGFs), bone morphogenic proteins (BMPs), retinoic acid (RA) and sonic hedgehog (SHH) [51]. During foregut patterning, it has been shown that bFGF specifies hESCs-derived definitive endoderm into different foregut lineages in a dose-dependent manner [52]. q-PCR experiments confirmed that treating cells with a combination of bFGF and RA is essential to induce an increase of *PDX1* expression. Nevertheless, this treatment also induces other foregut-derivate endoderm as observed by the strong peak of expression of *HNFs* genes on day 8–11. In early development, SHH is highly expressed in stomach and duodenal endoderm, but not in pancreatic endoderm. Accordingly, a specific inhibition of SHH signaling has been shown to promote *in vitro* pancreatic differentiation by blocking stomach and duodenal endoderm formation [5,53]. In the same way as inhibition of SHH avoids stomach and duodenal endoderm specification, the inhibition of BMP signaling pathway by Noggin has been shown to block the hepatic commitment [47]. On this basis, we used a sequential addition of Noggin and Cyclopamine to promote pancreatic endoderm specification at the expense of other foregut endoderm lineages. We demonstrated that this combination of factors resulted in a very effective pancreatic specification as evidenced by both a drastic down-regulation of *HNFs* genes and a strong up-regulation of the pancreatic precursors *NGN3* and *NKX2*.*2* genes. To obtain a further differentiation towards an endocrine fate, we promote the re-aggregation of cells in the form of an islet-like three-dimensional structure by culturing them under suspension culture as previously described by Shim et al [36]. The last step of our differentiation protocol consists on directing the maturation of hESCs-derived endocrine precursors toward specialized and functional hormone-secreting cells. Actually, insulin-producing cells obtained by the numerous *in vitro* differentiation protocols published so far are commonly immature and non-functionally glucose-responsive [20]. As a consequence, many research groups omitted the late *in vitro* differentiation steps, and allowed pancreatic progenitors to specialize into functional β-cell by *in vivo* maturation after transplantation in STZ-induced hyperglycaemic mice [12,14–16,54] or included small molecules and growth factors to medium formulation in the last stage of cell differentiation. IGF1, Exendin-4, HGF and B27-supplement were used as a “maturation factors” during late differentiation stages, but only minor effects were observed [35], and recently, R428 (a selective small-molecule inhibitor of the tyrosine kinase receptor AXL), Alk5 receptor inhibitor (Alk5i), N-acetyl cysteine (N-Cys) and thyroid hormone T3 were successfully used to come up with highly differentiated cells quite similar to mature β-cells [12,13]. In our hands and in addition to RSV, fibronectin and insulin-transferrin-selenium (ITS) were used during the suspension culture step as previously reported [36]. Characterization of the cells derived by the application of this differentiation protocol confirmed that we were able to obtain islet-like clusters that fully express different markers of a mature Langerhans islet. RT-PCR analysis could confirm the expression of several pancreatic endocrine hormones (insulin, glucagon, and somatostatin) and, more importantly, the expression of different markers of a mature β-cell *GLUT2*, *GK*, *KIR6*.*2*, *PC1/3*, *PC2* and *INS*. Almost the same results were obtained when the differentiation protocol was applied to a different type of stem cells, the hiPSC line MSUH001. Similarly, insulin and MAFA positive cells were generated with hiPSC, but with reduced efficiency [12]; thus suggesting that further optimizations are required when other pluripotent cells are used.

Finally we demonstrate the effects of RSV on these insulin-producing cells demonstrating that, like observed in INS-1E cells, RSV was able to increase insulin content and secretion of hESCs-derived β-cell-like cells. Surprisingly our results with hESCs were even more effective. Indeed, RSV not only increased the amount of insulin secretion but it was also able to ameliorate the maturation process. Differentiation protocols incorporating RSV treatment yielded typically 40% of insulin positive cells a similar achievement as reported by Rezania et al and Pagliuca et al [12,13] and almost 50% more than previous published *in vitro* differentiation protocol. We achieved a higher percentage of insulin positive cells, and an increase of both insulin production and secretion. Moreover, differentiated cells were able to complete the maturation process and be functional when transplanted in diabetic mice as observed in the *in vivo* experiments. The rapid reversion of hyperglycaemia observed in transplanted mice confirmed the advanced grade of commitment of our cells toward an almost mature and functional phenotype. Importantly, we did not observe any tumor formation in our NOD-SCID transplanted mice.

The beneficial effects of RSV on hESCs-derived β-cell-like cells differentiation are probably mediated by the up-regulation of *PDX1* expression, a mechanism that was previously demonstrated on INS-1E cells, non-human primates and human islets [25,33]. By inducing *PDX1* expression and others β-cell markers, RSV has been shown to exert different effects in different cells: it can maintain the β-cell identity by preventing existing β-cell dedifferentiation [33] or it can induce a combination of β-cell genes into a non-β-cell background such as α-cells [34] or as in our case, β-cell precursors. Nonetheless, the mechanism by which RSV induced the increase of *PDX1* expression was not clear. We hypothesized that it could be induced through the activation of the signaling pathways upstream of *PDX1* expression. RSV has been shown to activate AMPK in different cell types [55–57] including β-cells [25]. Moreover, it has been recently shown that RSV activates AMPK in a SIRT1-dipendent way at low concentrations or in a SIRT1-independent way at higher RSV concentrations [58]. We studied AMPK phosphorylation in hESCs-derived insulin secreting cells and observed an increase of phosphorylated AMPK in cells treated with RSV, this finding was consistent with the results published by Vetterli et al [25]. In addition to the activation of the AMPK signaling pathway there is another cascade of kinases upstream of *PDX1* that is involved in its transcriptional activation, the PI3K/AKT signaling pathway [59,60]. Our results indicate that RSV also acts through the activation of the PI3K/AKT signaling pathway. We propose that the activation of both the AMPK and PI3K signaling pathways is the main mechanism by which RSV increased *PDX1* expression which is mandatory to induce the expression of downstream genes involved in the correct functionality of a mature β-cell. In summary we demonstrated that RSV not only improved glucose-stimulated insulin secretion in differentiated β-cells but also triggered maturation of pancreatic endocrine precursors towards a β-cell phenotype through the activation of *PDX1* gene. We are aware that much work remains to be done, but this approach represents a step forward for future cell therapy of diabetes mellitus, we also should keep in mind the safety and tolerability of RSV [61], and its promise for improvement of general health of diabetic patients demonstrated by on-going clinical trials [62–64].

## Materials and Methods

### Cell culture

The hESC line HS-181 was derived in the Fertility Unit of Karolinska University Hospital, Huddinge at the Karolinska Institute after approval of a project entitled “Derivation and early differentiation and characterization of hESC lines” by the Karolinska Institute Research Ethics Board South, Drno 454/02. This line was derived from an embryo that could not be used for the infertility treatment of a couple. Both partners of the couple signed a consent form for donation of the embryo for derivation of a possible permanent stem cell line to be used in stem cell research. The HS-181 line is included in the European Union hESC registry (http://www.hescreg.eu/) and cultured as described by Hovatta et al [65]. Briefly, HS181 was cultured onto BD Matrigel hESC-Qualified Matrix coated flasks (BD Biosicences, San Diego, CA, USA), in human feeder-conditioned medium consisting of knockout DMEM (Gibco, Grand Island, NY, USA), 20% serum replacement (SR) (Gibco), 2 mM L-glutamine (Gibco), 1% non-essential amino acids (Gibco), 50 U/ml penicillin (Gibco), 50 μg/ml streptomycin (Gibco), 0,1 mM β-mercaptoethanol (Gibco) and 4ng/ml basic fibroblast growth factor (bFGF) (R&D Systems, Minneapolis, MN, USA). The hiPSC line MSUH-001 was obtained from the ISCIII National Biobank (Spanish Ministry of Health) and cultured as described for the HS-181 cell line.

INS-1E insulinoma cells are subclones of parental INS-1 cells [66], these cells were generously provided by F.J. Bedoya (CABIMER Sevilla, Spain), INS-1E cells were seeded at 3x10^5^ cell density and cultured in a humidified atmosphere (5% CO2) in RPMI-1640 medium (Lonza, Basel, Switzerland) supplemented with 10% fetal bovine serum (Lonza) and 11.1 mM glucose (Sigma Aldrich, St. Louis, MO, USA), 1 mM pyruvate (Gibco), 10 mM HEPES (Gibco), 2 mM L-glutamine (Gibco), 50 μM β-mercaptoethanol (Gibco), 100 U/ml penicillin (Gibco) and 100 μg/ml streptomycin (Gibco). Cells were passed weekly with trypsin-EDTA treatment. For each independent experiment cells were cultured during three days in growing media and then were exposed during 48 hours to different concentration of resveratrol (RSV) (Sigma Aldrich) or sirtinol (SRT) (Sigma Aldrich).

### In vitro differentiation of hESCs and hiPSCs

Differentiation of hESCs and hiPSCs was carried out in a step-wise differentiation protocol as illustrated schematically in Fig. 2B; briefly, 100 ng/ml activin A (R&D Systems) and 25 ng/ml Wnt3a (R&D Systems) were added to RPMI 1640 GlutaMax (Gibco) at day 1, in absence of serum, to induce mesendoderm; then fresh activin A was added daily and medium was changed to 0,2% fetal bovine serum (Lonza) during the second day and to 2% serum during the subsequent three days. Pancreas commitment was triggered by the addition of retinoic acid (RA) 2 μM (Sigma Aldrich), as a pancreatic morphogen inducer, and bFGF 64 ng/ml (R&D Systems) during three days. Subsequently 50 ng/ml noggin (R&D Systems) was added to the culture during three days to promote pancreatic fate and 0,25 μM cyclopamine-KAAD (Merck KGaA, Darmstadt, Germany) was used during the following three days to inhibit sonic hedgehog, a procedure that has been shown to increase *Pdx1* expression in mouse islets [5]. At this point cells were mechanically dissociated and passed from adherent culture to suspension culture in order to promote the three-dimensional islet-like cluster formation. The suspension culture was maintained in insulin-transferrin-selenium (ITS) medium (Gibco) containing 5 μg/ml fibronectin (Sigma Aldrich) and supplemented culture medium (Knockout DMEM 12% SR) during 6 days. Finally, to further promote the maturation of the β-cell phenotype, RSV 75 μM (Sigma Aldrich) was added to the suspension culture during the last two days of differentiation.

For spontaneous differentiation, hESCs and iPSCs were cultured during 22 days in RPMI 1640 medium with 0% FBS for the first day, 0.2% FBS during the second day, 2% FBS until day 14 and finally were passed in suspension culture with Knockout DMEM 12% SR medium till day 22.

### Immunofluorescence assay

INS-1E cells or hESCs-derived β-cell-like cells were fixed for 20 min in fresh 4% paraformaldehyde solution (Sigma Aldrich), washed three times with PBS and then permeabilized for 1 hour with PBS containing 0.5% Triton-X 100 (Sigma Aldrich). After 1 hour of blocking incubation with PBS supplemented with 4% bovine serum albumin (BSA) and 0.3% Triton-X 100, cells were incubated with primary antibodies over night at 4°C. Cells were then washed three times with PBS and incubated with secondary antibodies during 1 hour at room temperature. Finally, following three more PBS washing, nuclei were counterstained in PBS containing 1 μg/ml Hoechst 33342 (Sigma Aldrich). Antibodies information is provided in S1 Table. Digital images were obtained using a Leica SP5 confocal microscope (Leica, Mannheim, Germany) or an Olympus IX71 inverted microscope (Olympus, Tokyo, Japan). Fluorescence signals of Insulin expression were quantified with Meta Imaging Software MetaMorph Offline version 7.5.1.0 (MDS Analytical technologies, Sunnyvale, CA, USA).

### ELISA assay

For INS-1E assays, after two days of RSV or SRT treatments, medium was removed and cells were washed three times with warm Krebs buffer (NaCl 129 mM, NaHCO_3_ 5 mM, KCl 4.8 mM, KH_2_PO_4_ 1.2 mM, MgSO_4_ 1.2 mM, CaCl_2_ 1 mM, HEPES 10 mM, BSA 0.1% at pH 7.4). Cells were then incubated with Krebs solution during one hour at 37°C and, at the end of incubation, cells were stimulated with Krebs solution supplemented with 2,7 mM or 16,8 mM glucose for 30 min at 37°C. For hESCs assays, spontaneous differentiated clusters and islet-like clusters obtained at day 20 of the differentiation protocol were plated onto matrigel-coated dishes. Differentiated islet-like clusters were divided into two sub-conditions: one was treated with RSV and the other one not. After 48 hours, cells were treated according to the protocol described above, being the glucose stimulation period of 1 hour instead of 30 minutes. Supernatants were collected and insulin secretion was measured using a commercially available human insulin ELISA (Mercodia AB, Uppsala, Sweden), according to manufacturer’s specifications.

### Measurements of intracellular Calcium influx

INS-1E cells were loaded with Fura-2AM 2 μM during 30 min at 37°C and following a 15 min wash quantitative changes in intracellular calcium (Ca^2+^) (F340/F380 ratio) were monitored using dual-excitation fluorescence imaging system (InCyt Im2, Intracellular Imaging, Cincinnati, OH, USA) as previously described [67]. Experiments were performed in 0 mM Ca^2+^ solution containing 120 mM NaCl, 4.7 mM KCl, 4 mM MgCl2, 0.2 mM EGTA, and 10 mM HEPES, and the Ca^2+^ influx was determined from changes in Fura-2 fluorescence after the addition of Ca^2+^ (2 mM). Changes in intracellular Ca^2+^ were expressed as a change in ratio (ΔRatio), which was calculated as the difference between the peak F340/F380 ratio after extracellular Ca^2+^ was added and its basal level immediately before Ca^2+^ addition.

### Chromatin Immunoprecipitation (ChIP) assay

ChIP assays were performed according to Upstate (Merck KGaA) protocol. ChIP data are the average of real-time PCR (q-PCR) quantifications from three independent experiments. Samples were immunoprecipitated overnight at 4°C with 2 μg of a polyclonal antibody specific for SIRT1 (Merck KGaA) and immunoprecipitated DNA was analyzed by q-PCR of a regulatory region in the *Ucp2* promoter previously described in Bordone et al [30]. q-PCRs were performed in an Applied Biosystems 7500 Real-Time PCR System, using the PerfeCTa SYBR Green SuperMix Low ROX (Quanta Biosciences, Gaithersburg, MD, USA) and primers designed to span rat Ucp2 promotor (S2 Table). ChIP was quantified relative to inputs using the ΔΔCt method.

### RT-PCR and q-PCR analysis

Total RNA was extracted with TRIzol Reagent (Invitrogen) according to the manufacturer’s instructions. cDNA was synthesized from 1 μg total RNA by using MMLV reverse transcriptase (Promega, Madison, WI, USA). RT-PCR was performed using the BioTaq DNA Polymerase (Bioline, London, UK) following the manufacturer’s protocol. The cycle conditions were as follows: 94°C for 3 min followed by 35 cycles (94°C denaturation for 30 s, 55–68°C annealing for 1 min, 72°C for 1 min), with a final incubation at 72°C for 10 min. q-PCR analysis was performed on ABI Prism 7500 system (Applied Biosystems, Foster City, CA, USA) using the SYBR Green PCR Master Mix (Quanta Biosciences). The expression level of each gene at every checkpoint was normalized to UBC as an endogenous control. All quantities were expressed as number of folds relative to the expression level at Day 0, using the ΔΔCt method. In the case of no expression at Day 0, the Ct was arbitrarily set as 40. Primers information is provided in S2 Table.

### Western blotting analysis

Cells from the final step of differentiation were lysed in RIPA buffer (Sigma Aldrich) supplemented with a protease inhibitor cocktail (Roche Diagnostics, Basel, Switzerland) and phosphatase (Sigma Aldrich) inhibitors during 45 minutes, sonicated and then centrifuged at 14000 rpm for 20 min at 4°C. The supernatant was removed and the amount of protein was determined by Bradford method using the Bio-Rad protein dye reagent (Bio-Rad, Hercules, Ca, USA), protein lysates were subjected to electrophoresis separation (30–40 μg/lane) in 10% SDS-PAGE and transferred to a Hybond-P polyvinylidene difluoride (PVDF) membrane (Amersham, Buckinghamshire, UK). Membranes were blocked overnight at 4°C in Tris-buffered saline with 5% BSA (Sigma Aldrich) and 0.1% Tween 20 (Sigma Aldrich). Blots were incubated overnight with primary antibodies and immunoreactive bands were detected with horseradish peroxidase-conjugated secondary antibodies followed by ECL Prime detection system (Amersham) and exposed to photographic film (Amersham). Primary and secondary antibodies used are listed in S1 Table. For bands optical density determination, photographic films were scanned using an ImmageScanner and LabScan software version 5.0 (Amersham), and the resulting images were quantified using the ImmageQuant software version 5.2 (Molecular Dynamics, Sunnyvale, CA, USA). The data were expressed as relative densitometry units.

### STZ treatment for diabetes induction

Male 8 weeks old NOD/SCID mice (NOD.CB17-Prkdc scid/J) were purchased from Charles River Laboratories (Charles River Laboratories International, Barcelona, Spain). Mice received a single intraperitoneal injection of STZ (Sigma Aldrich) 170 mg/Kg freshly dissolved in citrate buffer (pH 4.5). Animals were considered diabetic when they reached blood glucose levels > 300 mg/dl for at least 3 consecutive days. Blood glucose was monitored daily from a tail blood sample and following 3 hours morning fast using ACCU-CHEK Compact Plus glucometer (Roche Diagnostics).

### In vivo cell Transplantation

The day of transplantation, hESCs-derived islet-like clusters from day 22 of the differentiation protocol were recollected and injected under the kidney capsule of diabetic mice. Briefly, mice were anesthetized with Ketamine (100 mg/kg) and Xylazine (10 mg/kg), the kidney was exposed through a lumbar incision and slurry of 10–20 μl of aggregates (~2000 islet-like clusters) was delivered under the kidney capsule with a catheter connected to a micropipette. Body weight and blood glucose level were monitored every two days as indicated above. Grafts were removed 4–6 weeks after transplantation, fixed with 4% paraformaldehyde solution overnight at 4°C, washed with PBS and transferred to a 30% sucrose solution over night at 4°C. Finally grafts were mounted in frozen blocks with Tissue-Tek O.C.T. compound (Sakura Finetek, Torrance, CA, USA) and were cut into 10 μm thick tissue sections using a cryostat. Frozen sections were subjected to immunohistochemistry or to haematoxylin/eosin staining. All mouse experiments complied with Institutional Animal Care and Use Committee guidelines of the Department of Agriculture and Fishery for the Regional Government of Andalucia (Directive 2010/63/EU). This study was carried out in strict accordance with the guidelines for animal research protocols established and approved by Animal Experimentation and Ethics Committee of CABIMER (CEEA-CABIMER). This Committee CEEA-CABIMER specifically approved this study (Permit Number: CEEA no.1/2012). All efforts were made to minimize animals suffering.

### Statistical analysis

Values are presented as mean ±SE. Statistical significance was calculated by using Student’s t-test, *p<0*.*05* was considered statistically significant.

## Supporting Information

S1 Table List of Antibodies used in this study. (PDF)

S2 Table Primer Sequence Sets. (PDF)
