# Supplementary material for: Resveratrol Ameliorates the Maturation Process of β-Cell-Like Cells Obtained from an Optimized Differentiation Protocol of Human Embryonic Stem Cells
Source: PLoS One. 2015 Mar 16;10(3):e0119904. doi: 10.1371/journal.pone.0119904 (PMC4361612; doi:10.1371/journal.pone.0119904)
Supplement: S1 Table — (PDF) [file pone.0119904.s001.pdf]

S1 Table. List of Antibodies used in this study

Primary Antibodies					
				Dilution	
**Antibody**	**Supplier**	**Reference**	**Source**	**IF**	**WB**
Monoclonal anti-INS*	Sigma Aldrich	I2018	Mouse	1:500	-
Polyclonal anti-INS	Dako	A0564	Guinea pig	1:500	-
Monoclonal anti-GCG	Sigma Aldrich	G2656	Mouse	1:500	-
Polyclonal anti-PDX1	Merk Millipore	AB3505	Rabbit	1:1000	-
Monoclonal anti-C-PEP	Merk Millipore	05-1109	Mouse	1:500	-
Monoclonal anti-AMPK	Cell Signal Technology	2793S	Mouse	-	1:1000
Monoclonal anti-pAMPK	Cell Signal Technology	2535S	Rabbit	-	1:1000
Polyclonal anti-PI3K	Cell Signal Technology	4292S	Rabbit	-	1:1000
Polyclonal anti-pPI3K	Cell Signal Technology	4228S	Rabbit	-	1:1000
Polyclonal anti-AKT	Cell Signal Technology	9272S	Rabbit	-	1:1000
Polyclonal anti-pAKT	Cell Signal Technology	9271S	Rabbit	-	1:1000
Polyclonal anti-MAPK	Cell Signal Technology	9102S	Rabbit	-	1:1000
Monoclonal anti-pMAPK	Cell Signal Technology	9106S	Mouse	-	1:1000
Monoclonal anti-β-actin	Sigma Aldrich	A5441	Mouse	-	1:10000

Secondary antibodies					
**Antibody**	**Supplier**	**Reference**	**Source**	**Label**	**Dilution**
Mouse IgG	Invitrogen	A11029	Goat	Alexa Fluor 488	1:500
Rabbit IgG	Invitrogen	A11034	Goat	Alexa Fluor 488	1:500
Mouse IgG	Invitrogen	A11032	Goat	Alexa Fluor 594	1:500
Rabbit IgG	Invitrogen	A11037	Goat	Alexa Fluor 594	1:500
Guinea pig IgG	Rockland Immunochemicals	606-142-129	Goat	DyLight 549	1:4000
Mouse IgG	Jackson ImmunoResearch	115-035-003	Goat	HRP (horseradish peroxidase)	1:20000
Rabbit IgG	Jackson ImmunoResearch	111-035-003	Goat	HRP (horseradish peroxidase)	1:20000

* This antibody has been used only for INS-1E immunofluorescence.
