# Supplementary material for: Resveratrol Ameliorates the Maturation Process of β-Cell-Like Cells Obtained from an Optimized Differentiation Protocol of Human Embryonic Stem Cells
Source: PLoS One. 2015 Mar 16;10(3):e0119904. doi: 10.1371/journal.pone.0119904 (PMC4361612; doi:10.1371/journal.pone.0119904)
Supplement: S2 Table — (PDF) [file pone.0119904.s002.pdf]

S2 Table. Primer Sequence Sets

RT-PCR Primer sets

Gene symbol	Forward primer sequence (5'-3')	Reverse primer sequence (5'-3')
SOX17	TGGACCGCACGGAATTTGAACA	TGTGTAACACTGCTTCTGGCCT
FOXA2	TTGCTGGTCGTTTGTTGTGGCT	TTCATGTTGCTCACGGAGGAGT
NGN3	TAAGAGCGAGTTGGCACTGAGCAA	TTTGAGTCAGCGCCCAGATGTAGT
ISL1	GTGCGGAGTGTAATCAGTATTTGG	GTCATCTCTACCAGTTGCTCCTTC
PDX1	TACTGGATTGGCGTTGTTTGTGGC	AGGGAGCCTTCCAATGTGTATGGT
NKX2.2	CGGACAATGACAAGGAGACCCCG	CGCTCACCAAGTCCACTGCTGCTGG
KIR6.2	CGCTGGTGGACCTCAAGTGGC	CCTCGGGGCTGGTGGTCTTGCG
SUR1	TGCACATCCACCACAGCACATGGCTTC	GTGTCTTGAAGAAGATGTATCTCCTCAC
GLUT2	GGTTTGTAACTTATGCCTTAAG	GCCTAGTTATGCATTGCAG
GK	GACGAGTTCCTGCTGGAGTATGAC	GACTCGATGAAGGTGATCTCGCAGCTG
PC1/3	TTGGCTGAAAGAGAACGGGATACATCT	ACTTCTTTGGTGATTGCTTTGGCGGTG
PC2	GCATCAAGCACAGACCTACACTCG	GAGACACAACCACCCTTCATCCTTC
INS	TTTGTGAACCAACACCTGTGCGG	TTGTTCCACAATGCCACGCTTCTG
GCG	CCAGATCATTCTCAGCTTCC	GGCAATGTTATTCCTGTTCC
SST	CGTCAGTTTCTGCAGAAGTCCCTGGCT	CCATAGCCGGGTTTGAGTTAGCAGATC
PP	CTGCTGCTCCTGTCCACCTGCGTG	CTCCGAGAAGGCCAGCGTGTCCTC
β-actin	CGTACCACTGGCATCGTGAT	TTCTCCTTAATGTCACGCAC

q-PCR Primer sets

Gene symbol	Forward primer sequence (5'-3')	Reverse primer sequence (5'-3')
*Ucp2**	CGTCTGTTCAAAGCGTCTCA	CCAGCTGGAGTCTTCTCCTT
SOX17	TGGACCGCACGGAATTTGAACA	TGTGTAACACTGCTTCTGGCCT
FOXA2	TTGCTGGTCGTTTGTTGTGGCT	TTCATGTTGCTCACGGAGGAGT
HNF1B	TCACAGATACCAGCAGCATCAGT	GGGCATCACCAGGCTTGTA
HNF4A	CATGGCCAAGATTGACAACCT	TTCCCATATGTTCCTGCATCAG

PDX1	TACTGGATTGGCGTTGTTTGTGGC	AGGGAGCCTTCCAATGTGTATGGT
NGN3	TAAGAGCGAGTTGGCACTGAGCAA	TTTGAGTCAGCGCCCAGATGTAGT
NKX2.2	GGCCTTCAGTACTCCCTGCA	GGGACTTGGAGCTTGAGTCCT
INS	TTTGTGAACCAACACCTGTGCGG	TTGTTCCACAATGCCACGCTTCTG
GLUT2	TTCCGGAATCAATGGCATTT	TGTTTACAGCGCCAACTCCA
GK	CCTGGGTGGCACTAACTTCAG	TAGTCGAAGAGCATCTCAGCA

* Primer sequences for rat *Ucp2* promoter.
